# Effectiveness of orofacial myofunctional therapy in orthodontic patients: A systematic review

**DOI:** 10.1590/2176-9451.19.4.094-099.oar

**Published:** 2014

**Authors:** Márcio Alexandre Homem, Raquel Gonçalves Vieira-Andrade, Saulo Gabriel Moreira Falci, Maria Letícia Ramos-Jorge, Leandro Silva Marques

**Affiliations:** 1 MSc in Dentistry, Federal University of the Jequitinhonha and Mucuri Valleys (UFVJM).; 2 PhD Resident in Dentistry, Federal University of Minas Gerais (UFMG).; 3 Visiting professor, Federal University of the Jequitinhonha and Mucuri Valleys (UFVJM).; 4 Adjunct professor, Department of Dentistry, Federal University of the Jequitinhonha and Mucuri Valleys (UFVJM).

**Keywords:** Myofunctional therapy, Orthodontics, Malocclusion

## Abstract

**Objective:**

The aim of the present systematic review was to determine the existence of scientific evidence demonstrating the effectiveness of orofacial myofunctional therapy (OMT) as an adjuvant to orthodontic treatment in individuals with orofacial disorders. A further aim was to assess the methodological quality of the studies included in the review.

**Methods:**

An electronic search was performed in eight databases (Medline, BBO, LILACS, Web of Science, EMBASE, BIREME, Cochrane Library and SciELO) for papers published between January 1965 and March 2011, with no language restrictions. Selection of articles and data extraction were performed by two independent researchers. The quality of the selected articles was also assessed.

**Results:**

Search strategy resulted in the retrieval of 355 publications, only four of which fulfilled the eligibility criteria and qualified for final analysis. All papers selected had a high risk of bias.

**Conclusions:**

The findings of the present systematic review demonstrate the scarcity of consistent studies and scientific evidence supporting the use of OMT in combination with orthodontic treatment to achieve better results in the correction of dentofacial disorders in individuals with orofacial abnormalities.

## Abstract (pt)

**Objetivo:**

o objetivo dessa revisão sistemática foi verificar se existem evidências científicas que comprovam a efetividade da TMO como agente coadjuvante do tratamento ortodôntico de indivíduos com distúrbios orofaciais. Além disso, avaliar a qualidade metodológica dos estudos incluídos nessa revisão.

**Métodos:**

uma busca eletrônica foi realizada em 8 bases de dados (MEDLINE, BBO, LILACS, Web of Science, EMBASE, BIREME, Cochrane Library e Scielo), sem restrição de idioma. A busca foi realizada com artigos publicados no período compreendido entre janeiro de 1965 a março de 2011. A seleção dos artigos e extração dos dados foi realizada por dois revisores independentes. Avaliação da qualidade dos artigos também foi realizada.

**Resultados:**

a estratégia de busca resultou em 355 publicações. Após seleção baseada nos critérios de elegibilidade, quatro artigos foram qualificados para análise final. A todos os artigos incluídos nessa revisão foram atribuídos um alto risco de viés.

**Conclusções:**

os resultados do presente estudo demonstram a escassez de estudos consistentes e de evidências científicas que indicam a utilização da TMO em associação ao tratamento ortodôntico com a finalidade de promover melhores resultados na correção de desordens dentofaciais em indivíduos com distúrbios orofaciais.

## INTRODUCTION

Orofacial myofunctional therapy (OMT) techniques and principles can be used either alone or in combination with other forms of therapy.^1-7^ In combination with Orthodontics, OMT has been reported to be effective in the treatment of myofunctional disorders.^2,5-11^ According to a number of studies, this combination leads to improvements in myofunctional capacity, allows satisfactory growth and development of the maxilla and assists in the adaptation of dentition to the new occlusal pattern.^8,12,13^ However, a critical literature analysis reveals that most studies on this topic have striking methodological differences, heterogeneous samples and a lack of representativity.^3^ Such limitations have led to divergent results and compromise the quality of evidence, thereby hindering interpretation and clinical application of findings.

OMT generally involves exercising the facial and cervical muscles to improve proprioception, tone and mobility.^1,14-18^ The main objectives are the treatment of disorders of the stomatognathic system, such as orofacial abnormalities, mouth-breathing pattern, lip incompetence, tongue thrust habit, mandibular deviation and improper joint patterns during speech; chewing and swallowing, as well as assistance in the correction of parafunctional oral habits, such as thumb-sucking and bruxism.^1,14-24^ In some cases, OMT may also assist in improving body posture, thereby contributing to overall health.^1,14-18^

Since orofacial disorders increase the degree of difficulty of orthodontic treatment and contribute to the relapse of dentofacial abnormalities,^8,9,11^ OMT may be favorable to orthodontic treatment. Although the literature reports the combination of these therapies to be fundamental to achieve a satisfactory outcome in orthodontic treatment, there have been no systematic reviews carried out to investigate whether this combination is truly capable of achieving better results regarding dentofacial disorders in individuals with orofacial abnormalities.

The aim of the present systematic review was to determine scientific evidence that confirms the effectiveness of OMT as a complement to orthodontic treatment in individuals with orofacial disorders. A further aim was to assess the methodological quality of the studies included in the review.

## MATERIAL AND METHODS

Eligibility criteria were defined by the authors prior to beginning the study. *In vivo* prospective, longitudinal studies and randomized and/or controlled clinical trials that evaluated the effectiveness of OMT combined with orthodontic treatment in healthy patients with dentofacial deformities were included in the review. Case reports, case series, review articles, opinions and *in vitro* studies were excluded. No restrictions were made with regard to language.

### Type of intervention

Orthodontic treatment combined with OMT in patients with malocclusions and/or deficiencies in the vertical, sagittal and transverse directions and/or orofacial dyskinesia.

### Search strategy

Searches were performed in the following electronic databases:

» BIREME - Latin American and Caribbean Center of Health Sciences (www.bireme.br).
» LILACS - Latin American and Caribbean Literature on Health Sciences.
» MEDLINE -Medical Literature Analysis and Retrieval System Online.
» Web of Science - Referential database with abstracts in the fields of science, social science, arts and humanities.
» Cochrane Library (http://cochrane.bvsalud.org) - database of papers with a high degree of scientific evidence, including systematic reviews, controlled clinical trials, etc.
» BBO - Brazilian Library of Dentistry.
» SciELO - Online Electronic Scientific Library.

A search was performed for articles published between January 1965 and March 2011, suing the following keywords: "myofunctional therapy", "oral myofunctional therapy", "orofacial myofunctional therapy", "myofunctional therapy effectiveness", "orthodontic treatment and therapy myofunctional", "myofunctional therapy and orthodontics". All these keywords were used in all the aforementioned databases.

### Selection criteria and data extraction

Three selection phases were carried out by two independent researchers, with differences in opinion settled by consensus. Initially, all titles were analyzed to eliminate irrelevant publications, review articles, studies involving animals and *in vitro* studies. All abstracts of the publications selected in the first phase were then analyzed and only those referring to prospective, longitudinal studies and randomized clinical trials were included. The full texts of the articles selected in the second phase were read and eligibility was based on the evaluation of effectiveness of OMT in combination with orthodontic treatment.

A table was constructed with data from all studies and the findings were discussed. The following data were recorded: author, year of publication, study design, study groups, sample, age, methods/measures and assessment of results. A high level of agreement between the two researchers was achieved in this phase.

### Quality assessment

Methodological quality of studies was assessed with a combination of criteria established by Moose^25^ and Prisma.^26^ The risk of bias was considered low when all the following criteria were reported: 1) randomized sample selection; 2) definition of inclusion and exclusion criteria for the sample; 3) declaration of losses during follow-up; 4) use of validated measures; and 5) adequate statistical analysis. When one of these criteria was absent, the risk of bias was considered moderate. When two or more criteria were absent, the risk of bias was considered high.

## RESULTS

**Figure 1:**
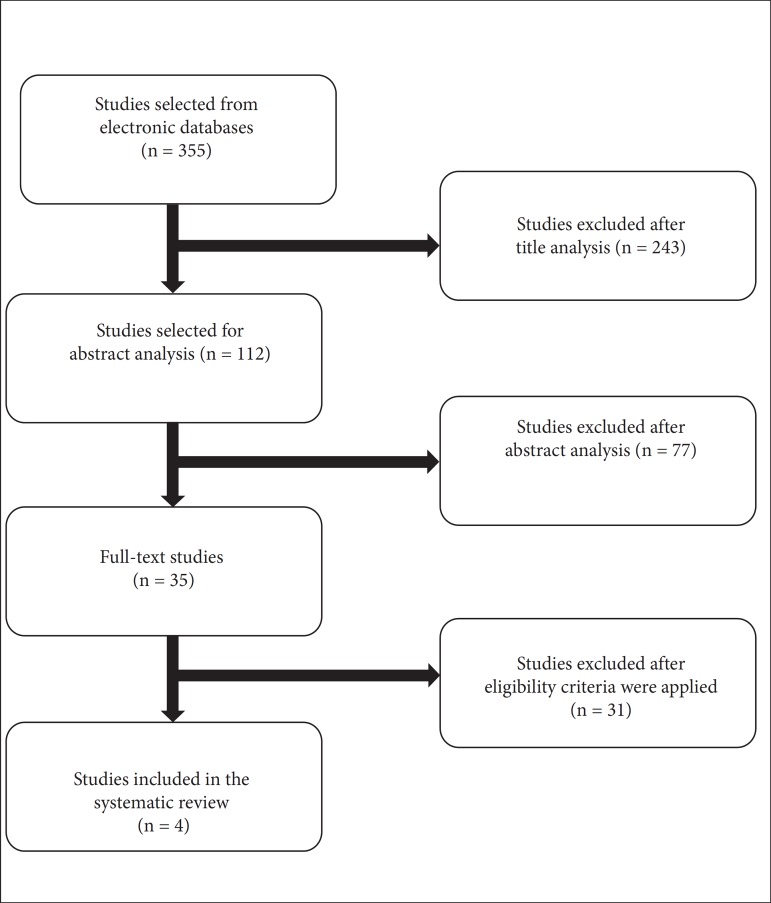
Flowchart of article selection process.

Search strategy resulted in 355 articles. Respecting all selection phases based on the eligibility criteria, four articles qualified for final analysis. Figure 1 displays the different steps of the selection process. Table 1 offers a detailed analysis of each article selected for the present systematic review.

**Table 1:** Characteristics of studies selected.

Author (year)	Study design	Study groups	Sample	Age	Methods/Measures	Assessment of results
Trawitzki et al^6^	LS	Experimental group:			Ultrasound of masseter muscle at rest and occlusion	Significantly greater (P < 0.01) masseter muscle thickness (cm) in P3 group
		*P1 (before surgery): patients with Class III malocclusion				
		*P3 (same patients 3 years and 3 years and 8 months after surgery):	13	21/42		
		Finalization of orthodontic treatment and OMT	15		Linear regression test	
		Control group: Individuals without morphological facial abnormalities				
Smithpeter and Covell Jr^2^	LS	Patients with anterior open bite		8/41	T-test Analysis of covariance	- Relapse was 0.5 mm in experimental group and 3.4 mm in control group (significant difference)
		Experimental cohort group:				
		Individuals who received orthodontic treatment or retreatment and OMT	27		Linear regression	
			49			- OMT combined with orthodontic treatment was more effective in closure and maintenance of closure of anterior open bite in comparison to orthodontic treatment alone
		Control cohort group: Individuals with history of orthodontic treatment with relapse of open bite			Correlation coefficient	
Daglio et al^5^	CCT, LS	Patients with malocclusions, deficiencies in vertical, sagittal and transverse dimensions and orofacial dyskinesis			Statistical homogeneity	- Group A: Reduction in overjet from 3.5 to 2.6 mm; angle of base of mandible reduced from 30° to 28.31°; ANB angle reduced from 4.4° to 2.7°; statistically significant changes; better results with correction of overbite, which was normalized from a mean of -2.46 to 3.06 mm
		Control group (A):	13	8/17		
		Treated with OMT alone	15		Payne test	- Group B: Reduction in overjet from 6.6 to 2.6 mm; overbite improved from mean of -1.2 to +2.9 mm; angle of base of mandible reduced from 31.2° to 27.8°; ANB angle reduced from 7.3° to 3.7°
		Experimental group (B): treated with combined OMT and orthodontic appliance			Frequency analysis	
Daglio et al^7^	CCT, LS		75	6/22	Payne test Homogeneity test	Combination of OMT and orthodontic treatment was more successful in correction of resting lip posture than OMT alone
		Patients with orofacial dyskinensia and anterior open bite				
		Experimental group:				
		OMT + orthodontic treatment			Cephalometric analysis	
		Control group:			Correlation analysis	
		OMT alone			Frequency analysis	


LS - Longitudinal Study, CCT - Controlled Clinical Trial.

### Quality of studies

All articles included in this review had a high risk of bias (Table 2). None of the papers selected presented information on randomized selection of the sample or definition of the inclusion and exclusion criteria.

**Table 2:** Quality assessment of studies selected.

Quality criteria	Trawitzki et al^6^	Smithpeter and Covell Jr^2^	Daglio et al^5^	Daglio et al^7^
Randomized sample selection	No	No	No	No
Definition of inclusion and exclusion criteria	No	No	No	No
Declaration of losses during follow-up	No	Yes	No	No
Use of validated measures	Yes	Yes	No	No
Adequate statistical analysis	Yes	Yes	Yes	Yes
Estimated potential risk of bias	High	High	High	High

## DISCUSSION

The present findings should be interpreted with caution, as only four papers met the eligibility criteria established and none exhibited a high degree of scientific evidence.^2,5,6,7^ Thus, while the studies selected indicated the efficacy of OMT in the correction of dentofacial disorders when combined with orthodontic treatment, the scarcity of consistent studies underscores the lack of scientific evidence on the actual effectiveness of OMT as a complement to orthodontic treatment.

From a methodological standpoint, all papers employed adequate statistical tests for data analysis.^2,5,6,7^ However, the considerable diversity of tests, together with the low number of studies included in the present review, impede carrying out a meta-analysis. Comparisons with other studies are also limited due to differences in study design, sample selection and sample size.

Two studies included in the present systematic review^5,7^ were carried out to determine the effectiveness of OMT alone (control group) and in combination with orthodontic treatment (experimental group). In one of these studies,^5^ the authors assessed patients with malocclusions, deficiencies in the vertical, sagittal and transverse dimensions and orofacial dyskinesia. Based on the findings, the authors report that patients with dyskinesia and malocclusions can be treated with both forms of therapy. In the other study involving only patients with orofacial dyskinesis and anterior open bite,^7^ the researchers found that the combination of OMT and orthodontic treatment was more successful in correcting lip incompetence than OMT alone. While their findings favor a combined therapeutic approach, the authors report that the decision regarding the use of OMT alone or in combination with orthodontic treatment is not conclusive and better planned studies are needed. Moreover, both studies have a high risk of bias and a substantial limitation, namely, that only one group was submitted to orthodontic treatment. Thus, the difference between groups is mainly related to the administration of orthodontic treatment.

Another article analyzed herein^2^ assessed the effectiveness of OMT as a complement to maintaining closure of anterior open bite following orthodontic treatment or retreatment. The main conclusion was that the relapse of open bite in the experimental group treated with both orthodontics and OMT (0.48 ± 0.8 mm) was significantly less than that in the control group treated with orthodontics alone (3.38 ± 1.3 mm) (P < 0.0001). Therefore, the authors indicate the combination of these two forms of therapy for anterior open bite and stress the importance of documenting the oral and functional habits of each patient, along with the traditional orthodontic records, in any study aimed at assessing the efficacy of treatment for open bite. Such an investigation would allow one to determine what kind of patients would benefit from the combination of OMT and orthodontic treatment and what kind of patients would have a good prognosis with the use of orthodontic appliances alone.

The most recent paper selected for this review^6^ assessed the effect of integrated treatment combining orthodontics, orthognathic surgery and OMT on the thickness of the masseter muscles in patients with Class III deformity. Although the study included orthognathic surgery as part of orthodontic treatment, the authors found that combined treatment with OMT and orthodontics led to an improvement in masseter muscle thickness in patients following orthognathic surgery in comparison to baseline and the control group. However, these findings should be interpreted with caution considering the high risk of bias as well as the fact that the difference between groups may have been related to the surgery itself, which was likely the main reason for the improvement in muscle thickness.

To reiterate, while the studies selected for the present systematic review indicate effectiveness of OMT in correcting dentofacial deformities when combined with orthodontic treatment, a number of limitations are found, especially with regard to the number and quality of the studies analyzed. Moreover, the papers investigated specific occlusal problems, such as anterior open bite, orofacial dyskinesia and masseter muscle thickness, which make the results quite particular to specific conditions. As one third of the population requires orthodontic treatment,^27^ further studies with more rigorous methods, such as randomized, controlled clinical trials, should be carried out to determine the actual effectiveness of OMT as a complement to orthodontic treatment.

## CONCLUSION

The findings of the present systematic review demonstrate a scarcity of consistent studies and scientific evidence supporting the use of OMT in combination with orthodontic treatment to achieve better results in the correction of dentofacial disorders in individuals with orofacial abnormalities. Studies with a high standard of quality and better study design are needed to establish strong scientific evidence that supports the indication of this form of combined therapy.
